# Sequencing of long stretches of repetitive DNA

**DOI:** 10.1038/srep36665

**Published:** 2016-11-07

**Authors:** Alfredo De Bustos, Angeles Cuadrado, Nicolás Jouve

**Affiliations:** 1 Department of Biomedicine and Biotechnology, University of Alcala, 28871 Alcalá de Henares (Madrid), Spain

## Abstract

Repetitive DNA is widespread in eukaryotic genomes, in some cases making up more than 80% of the total. SSRs are a type of repetitive DNA formed by short motifs repeated in tandem arrays. In some species, SSRs may be organized into long stretches, usually associated with the constitutive heterochromatin. Variation in repeats can alter the expression of genes, and changes in the number of repeats have been linked to certain human diseases. Unfortunately, the molecular characterization of these repeats has been hampered by technical limitations related to cloning and sequencing. Indeed, most sequenced genomes contain gaps owing to repetitive DNA-related assembly difficulties. This paper reports an alternative method for sequencing of long stretches of repetitive DNA based on the combined use of 1) a linear vector to stabilize the cloning process, and 2) the use of exonuclease III for obtaining progressive deletions of SSR-rich fragments. This strategy allowed the sequencing of a fragment containing a stretch of 6.2 kb of continuous SSRs. To demonstrate that this procedure can sequence other kinds of repetitive DNA, it was used to examine a 4.5 kb fragment containing a cluster of 15 repeats of the 5S rRNA gene of barley.

Repetitive DNA is present in most organisms in variable proportion; in some it is actually the most abundant component of genomic DNA^1^. There are three main classes of repeat: transposable elements, tandem repeats, and high copy number genes (such as ribosomal genes). Microsatellites, or simple sequence repeats (SSRs), are a type of repeat made up of units of up to 6 bp arranged in tandem arrays of very variable size distributed throughout the genome^2^. Given their high degree of polymorphism, SSRs have been widely used as molecular markers in mapping, DNA fingerprinting and genetic evolution studies. These sequences have also aroused attention given their apparent involvement in the onset of some heritable human disorders^3^. Most studies have relied on the analysis of SSRs located in coding regions of euchromatin in which the size of the repeats sequenced is around 100 bp. However, fluorescent *in situ* hybridisation (FISH) experiments have shown that some plant SSRs loci are organized into large clusters, usually associated with the constitutive heterochromatin^4^. These repetitive regions are poorly analyzed even in model organisms such as *D. melanogaster*, the genome of which is commonly regarded as completely sequenced^5^.

Failure to assemble long stretches of repeats accounts for most of the gaps that remain in “sequenced genomes”, including the human genome^6^. Indeed, even though next-generation sequencing (NGS) has provided vast amounts of genomic sequence data, many genomes are not fully known because of the difficulty in assembling repeated elements^7^. The sequence reads obtained with NGS platforms are short and simply do not span long repetitive sequences. In addition, numerous copies of reads can be nearly identical, leading to a tendency to assemble them into single, collapsed contigs. New bioinformatic strategies are under development to solve these problems^8^ but so far these have found the assembly of long stretches beyond their capacity. Another problem is the extreme difficulty in cloning highly repetitive DNA into plasmid vectors^9^. Fragments rich in repeats affect the structure of DNA, forming hairpins and other complex structures that induce instability^10^. Moreover, the superhelical stress induced by the presence of repeats generates secondary structures that become substrates for deletion. Parts of fragments containing repetitive DNA are therefore commonly lost during cloning^11^.

Our group has used SSRs as markers for the molecular characterization of chromosomes belonging to species of the family *Triticeae*, including wheat, barley and rye^12^,^13^. FISH experiments using SSRs as probes have allowed chromosome identification and the study of chromatin organization in wheat, the construction of physical maps of barley chromosomes, and the identification of rye chromosomes in several species of the genus *Secale*. Recently, phylogenetic studies using these markers in diploid and polyploid species of the genus *Hordeum* allowed the production of karyotypes that helped decipher relationships between homeologous chromosomes, thus contributing to our knowledge of species evolution in *Hordeum*^14^,^15^. The results permitted the identification of every chromosome in different taxa, and in some cases revealed the connection between polyploids and their potential diploid progenitors.

Our group is interested in understanding the molecular structure of SSRs in long stretches of repeats within the heterochromatin regions of the genomes of cereals. Repetitive DNA in heterochromatin can change rapidly in nucleotide sequence or copy number during evolution. However, this fraction of the genome is little understood in many species including barley, and it is considered essential for the epigenetic maintenance of centromeric function as well as for other cellular, developmental and evolutionary processes. In addition to exiting approaches, a strategy is here reported that allowed the sequencing of a 7.3 kb DNA fragment of the barley genome made up of SSRs motifs and could represent an alternative method for sequencing of long stretches of repetitive DNA. To demonstrate the value of this strategy, the sequence of a 4.5 kb fragment containing repeats of the tandem repetitive 5S rRNA gene of barley was sequenced. The method relies on the use of linear plasmids to clone fragments rich in SSRs motifs, and digestion with exonuclease in controlled serial deletions.

## Results and Discussion

The screening of a BAC library containing the barley genome using oligo (ACT)_5_ as a probe revealed several candidate clones rich in SSRs repeats (data not shown). To verify the presence of repeats, these clones were characterized by digesting them with different restriction enzymes which have no specific recognition sites within the repeat sequences analysed, allowing the identification of long SSR. In some cases the digestions showed smeared restriction signals in agarose gels, a previously described consequence of BAC clone instability^11^. The fragments obtained were hybridized with the same oligo (data not shown). For each digestion, several fragments of different size showed strong hybridization signals. A selection of fragments larger than 4 kb was used in subcloning and sequencing. Subcloning employing standard circular vectors was attempted, but the subclones obtained were highly unstable, hindering further experiments. A major drawback was the loss of repetitive DNA during the storing of the bacterial strain at −80 °C. In many cases, plasmid DNA obtained from different bacterial cultures of the same stored subclone rendered differences in size; in some cases the entire repetitive region was lost. This kind of loss has been related to the instability of the cloned region caused by the formation of secondary structures among the tandem repeats in the clones^9^. In addition, the features of typical cloning vectors increase this instability^16^, e.g., the promoters present in the vector backbone might express cloned DNA causing the rearrangement or deletion of the repeated sequences.

**Figure 1:**
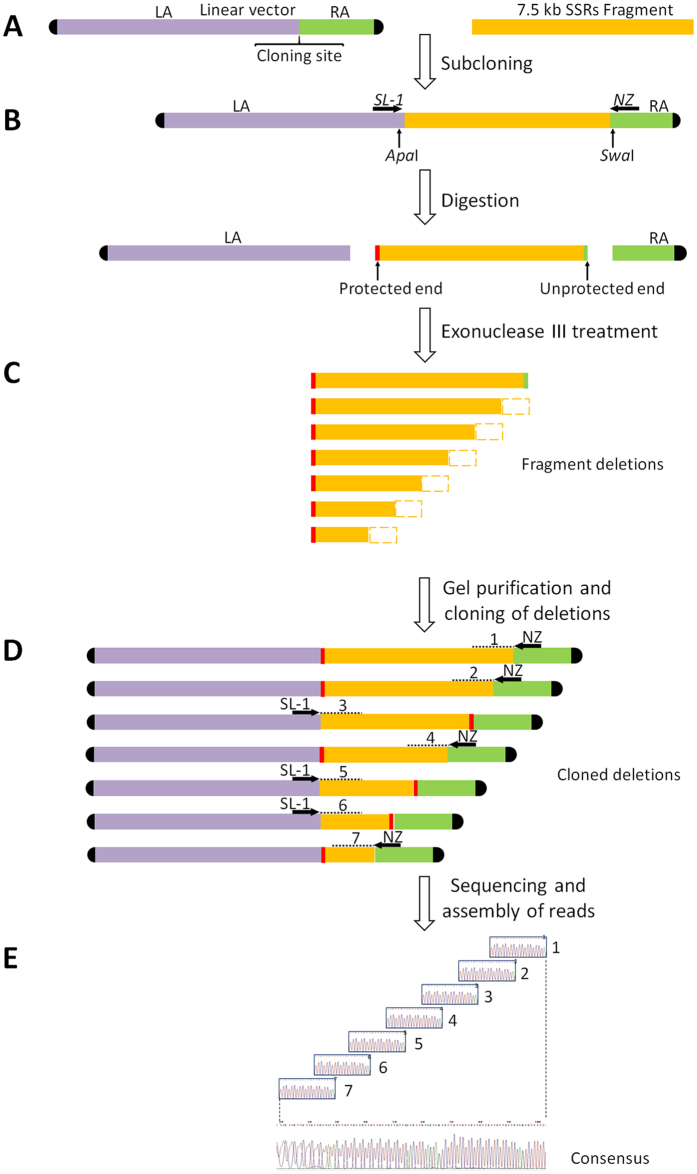
Procedure for the sequencing of repetitive DNA. (**A**) The pJAZZ linear vector was used for subcloning fragments rich in SSRs repeats. LA: left arm 10.3 kb; RA: right arm 2.2 kb. (**B**) Ends of fragments were sequenced using vector primers SL-1 and NZ. 3′ overhang and 5′ protruding ends were obtained by digesting the fragment with *Apa*I and *Swa*I restriction enzymes respectively. (**C**) Progressive unidirectional deletions produced by the digestion with exonuclease III after different times. The white box at the end of each deletion represents the portion of the fragment degraded in each consecutive interval. Note that during exonuclease treatment the vector is degraded. (**D**) Cloning of deletions into linear vector after purification from agarose gel and selection of a set of clones containing progressive deletions covering the size of the 7.5 kb fragment. Note that during the process fragments can be cloned in both orientations. For sequencing, SL-1 or NZ primers were used depending on the non-protected end of the deletion. Dotted lines indicate the read obtained from each clone; numbers indicate the order of assembly. (**E**) Obtaining the consensus sequence representing the entire 7.5 kb fragment via the assembly of ordered reads.

To overcome these problems, fragments rich in SSRs were subcloned using a linear vector (Fig. 1A). This sort of vector has been shown useful in stably maintaining tandem repeat inserts of up to 30 kb^9^; they also contain transcriptional terminators that prevent the expression of repeats. This enabled the cloning and manipulation of all fragments selected up to 20 kb in length (data not shown). The presence of repeats in these fragments was confirmed by the sequencing of the ends of the subclones using primers based on the pJAZZ vector (Fig. 1B). A subclone containing a fragment of around 7.5 kb rich in SSRs was selected to be entirely sequenced.

Attempts to sequence this fragment by the consecutive design of primers failed due to the inability to find unique specific sites for annealing. A strategy using exonuclease III was thus followed, based on previous work^17^. This relies on the production of consecutive, unidirectional deletions of the fragment by the exonuclease, and the posterior sequencing of these using vector primers. Exonuclease III removes nucleotides at the 3′ end of blunt or recessed ends and nicks although 3′ overhangs longer than 4 bases prevent the action of this enzyme^18^. The creation of 3′ overhangs at one end and 5′ protruding or blunt end at the other end of the fragment ensures deletion occurs in one direction only. For the present motif, a preliminary assay was performed to select the restriction enzymes that generated these kinds of end in the selected subclone. A protective *Apa*I unique restriction site was found in the vector 8 bp upstream of the fragment, and a *Swa*I blunt unique site located 87 bp downstream of the fragment (Fig. 1B).

After digestion of the subclone with both restriction enzymes, three aliquots of 5 μg each were treated with exonuclease III over different time intervals to generate progressive deletions of the fragment. The first aliquot was treated for 2 min and the remainder for 90 s longer each time. The digestion rate of exonuclease III at 37 °C is 450 bp/min^19^, therefore consecutive deletions of some 700–900 bp must be expected in each aliquot. However, in our experience, a mixture of fragments with different deletions, ranging from a few bases to more than 2000 bp is obtained in each aliquot. Thus, the exonuclease III digestions of the three aliquots generated enough deletions of some 400 bp to cover the entire fragment (Fig. 1C). During the exonuclease treatment the vector is degraded because the covalently closed ‘hairpin’ ends are not protected^9^.

Cloning of the fragments with deletions into the linear vector is necessary to multiply the DNA of interest so there is sufficient to be sequenced, and to avoid the presence of post-exonuclease treatment remains of vector fragments. The treated aliquots were therefore electrophoresed in an agarose gel and the fragments purified and cloned (Fig. 1D). An alternative method was also tried, in which it was sought to purify entire fragments from the agarose gel after the digestion with the two restriction enzymes but before the treatment with exonuclease III. However, digestion failed. The latter enzyme is highly sensitive to NaCl (concentrations as low as 20 mM have been known to affect its activity^19^, and the buffers supplied in agarose purification kits contain high NaCl concentrations. Despite repeated washes with 70% ethanol, this NaCl may have stalled all exonuclease III activity. Several more procedures were tried too, but these yielded only very low DNA concentrations preventing any further experiments.

**Figure 2:**
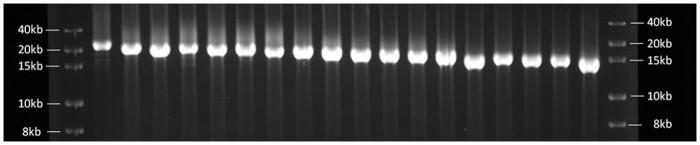
Agarose gel electrophoresis of several clones containing consecutive deletions of the fragment rich in SSRs. The first lane after the molecular marker (left) shows the clone with the original length fragment. Clones with differences in size of around 400 bp between consecutive deletions were selected. Clone size ranged from around 20 kb to 13 kb. Size of vector = 12.5 kb.

Clones containing deleted fragments were analysed by the toothpick plasmid assay^20^. This allowed the size of the insert to be estimated directly in the bacterial colonies by gel electrophoresis without the need for clone purification. This method is very effective when many clones need to be analyzed. Moreover, the use of a linear vector facilitated the estimation of clone size compared to the use of standard circular plasmids. Given the large size of some clones (up to 20 kb), overnight electrophoresis in agarose gels was performed. This led to the identification of bacterial colonies containing cloned fragment deletions with size differences of around 400 bp. A set of clones covering the complete original fragment were selected and purified (Fig. 2). The sequencing of these clones was performed using primers designed on the vector arms. In each case, the primer next to the non-protected end was used (Fig. 1D). Since the fragment deletions can be inserted in either direction during the cloning procedure, tests must be performed in order to select the correct primer. In this work, the digestion of clones with *Sfi*I produced a banding pattern that clearly identified the orientation of the cloned insert.

**Figure 3:**
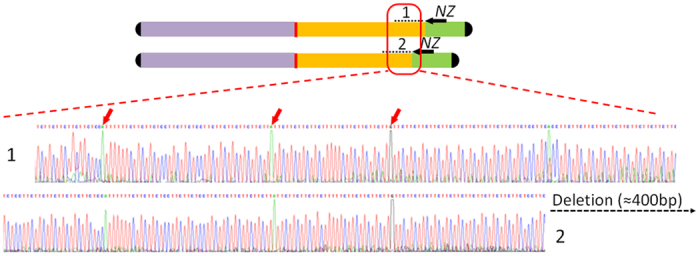
Assembly of reads from consecutive deletions. Precise overlapping is determined by the size of the deletion and single base changes of the repeated motif (red arrows).

**Figure 4:**
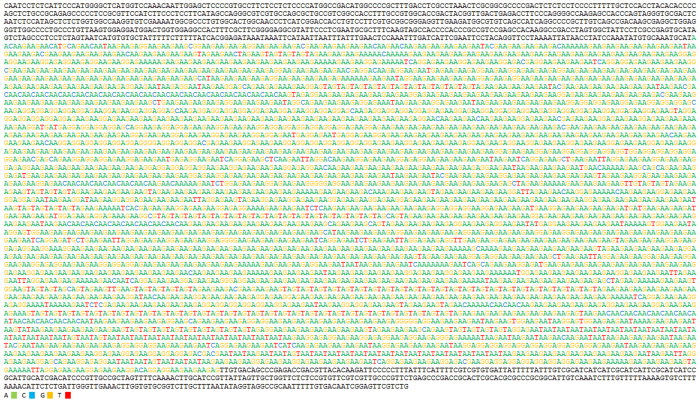
Consensus sequence of the fragment rich in SSRs. Variable numbers of repeats of trinucleotides AAG, AGT, AAC, AAT and AGG are interspersed in an uninterrupted region of around 6.2 kb. Bases of repeated regions have been coloured for easy identification of the repeats. GenBank accession number KX279876.

Sequencing of the selected clones provided a set of reads. To build the complete sequence of the original 7.5 kb fragment, reads were assembled following the order in which the fragment deletions were obtained (Fig. 1E). The reads from consecutive deletions with differences of around 400 bp were assembled (since Sanger sequencing usually produced reads of over 500 bp, the last 100 bp at least overlapped for consecutive reads). Assembly was facilitated by the presence of single base changes present in the overlapping region of consecutive reads (Fig. 3). Such nucleotide differences increase end-user confidence when matching up overlaps^8^. In the present work, although the sequencing of 20 clones containing consecutive deletions of 400 bp was sufficient to obtain the complete sequence of the 7.5 kb fragment, more than one clone was sequenced over the same part of the fragment in order to improve the reliability of the results. No differences were ever detected between clones of the same region, supporting the method’s reliability. The reads were assembled using CodonCode Aligner software, but most had to be manually assembled since the program was unable to resolve ambiguities and to overlap reads in the correct order. Manual assembly of more than 40 reads of continuous trinucleotide repeats was easily performed if they were assembled in the same order in which deletions were produced. In all cases, a few single changes in the repeat motif was enough to unambiguously assemble the reads. The consensus sequence for the assembled reads was a fragment of 7333 bp, with a region of trinucleotide repeats of around 6.2 kb (Fig. 4). In the hypothetical case of perfect repeats (never observed in the present study), assembly can be correctly performed if the exact size of deletion is known. This could be done by analyzing the size of the deleted fragments using an automatic fragment analyzer.

**Figure 5:**
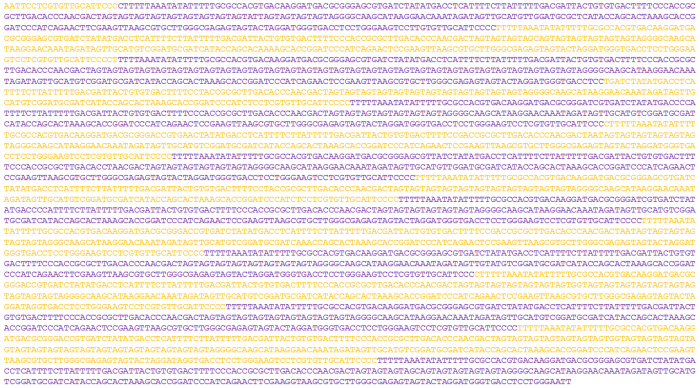
Sequenced DNA fragment containing several tandem arranged copies of the barley 5S sRNA gene. Tandem copies are shown in alternative pink and orange. GenBank accession number KX359334.

To show that this procedure can also be used to sequence repeated DNA sequences other than SSRs, a fragment of 4.5 kb containing the 5S rRNA gene was sequenced. In barley, 5S rRNA is produced from clusters of repeated genes^21^ - maybe thousands of copies - on chromosome arms 2HL, 3HL, 4HL and 7HS. Every copy contains a coding region of approximately 120 bp and a non-transcribed spacer (NTS) region of 100–700 bp^22^. This fragment was sequenced following the methodology described above, using *Apa*I and *Not*I enzymes to respectively generate the protected and non-protected ends in the fragment. The orientation of the cloned deletions was determined using the *BssS*I restriction enzyme. Reads were assembled in strict accordance with the order in which the deletions were produced, resulting in a consensus sequence of 4585 bp (Fig. 5) composed of 15 copies of the 5S rRNA gene.

Analysis of the molecular structure of repeated DNA has been hampered because of technical difficulties relating to the sequencing of repeats. In a pioneering attempt to analyze the molecular structure of SSRs, Ananiev *et al*.^11^ sequenced fragments no longer than 700 bp using traditional cloning and sequencing strategies, although fragments of more than 2 kb rich in SSRs were detected in Southern blot experiments. Technologies developed for the sequencing of complete genomes have also failed because they incorrectly assemble the reads for repeated regions. Modern 3rd-generation sequencing technologies such as single-molecule real-time (SMRT) sequencing using the PacBio platform can obtain reads of up to 2.3 kb of 100% repeated DNA^3^, but the error rate is relatively high^23^. Further, this procedure depends on the number of SMRT cells used^24^. Nanopore sequencing technologies can produce reads of adequate length (tens of kb or longer), but the accuracy of the technique needs to be greatly improved^25^. An example of the difficulty of sequencing this kind of DNA is showed in the last release of the reference sequence of the *Drosophila melanogaster* genome^5^. After most of one decade, the highly repeat-rich regions, including large satellite blocks and the ribosomal RNA genes and the centromere regions, are still inaccessible to current sequencing and assembly methods and remain poorly represented. For this reason, new sequencing technologies that produce very long reads of repeated DNA are required.

The proposed procedure provides an alternative method for sequencing of long stretches of repetitive DNA in addition to exiting approaches, and may be useful for the in-depth analysis of this type of DNA. The use of linear vectors overcomes the problems associated with the stability of these sequences during cloning. In addition, the serial production of deletions with exonuclease III allows the correct assembly of reads, overcoming the problems derived from their overlapping. This method could be of use in the analysis of specific repeated DNA such as that found in centromere or telomere sequences or to characterize genes with a repetitive structure. The proposed method is currently being used to sequence long stretches of other SSRs.

## Materials and Methods

### Barley BAC library screening and clone characterization

A total of 115,200 clones of the HVVMRXALLhB BAC library of barley were screened at the French Plant Genomic Resources Centre (INRA-CNRVG) at Toulouse (France). Radioactively labelled oligo (ACT)_5_ was used as a probe. Clones with strong signals were selected and their DNA purified using the NucleoBond Xtra BAC kit (Macherey-Nagel).

Southern blot experiments were conducted to characterize the clones. One microgram of DNA of each clone was digested with *EcoR*I, *Hind*III, *BamH*I, *KpnI, Bgl*II and *Sal*I restriction enzymes. Fragments were electrophoresed on 0.8% agarose gels and transferred to a nylon membrane. Hybridization was performed using digoxigenin-labelled (ACT)_5_ oligo as a probe. Positive fragments were subcloned into a *Sma*I site (blunt) on the pJAZZ linear vector (Lucigen) using the BigEasy v2.0 Linear Cloning Kits (Lucigen) following the manufacturer’s instructions. End sequencing of each subclone was performed using the vector primers SL-1 and NZ provided with the kit.

### Exonuclease digestion of subclones and sequencing

Two enzymes with unique restriction sites (one of them leaving a 4-base 3′ overhang) were selected to digest 30 μg of DNA of the subclones, each cutting at different ends of the fragment. The digested DNA was then purified using the DNA Clean & Concentrator-5 kit (Zymo Research). Aliquots of 5 μg were treated with 500 U of exonuclease III (Promega) at 37 °C for different lengths of time. The first was incubated for 2 min and then treated with 60 U of S1 Nuclease (Promega) for 30 min at room temperature to remove single-stranded tails. The reaction was then stopped by adding 1 μl of stop solution (300 mM Tris base, 50 mM EDTA) and incubating at 70 °C for 10 min. The remaining aliquots were incubated with exonuclease over intervals increasing each time by 90 s, before being subjected to the rest of the treatment. The reaction products were run in 0.8% agarose gels and the digested fragments purified using the Speedtools PCR Clean Up kit (BioTools). Each fragment was end-repaired using the DNATerminator^®^ End Repair kit (Lucigen) and cloned into the pJAZZ vector as described above. Clones containing fragments were selected by the toothpick assay^20^, inoculating 1 ml of LB medium with appropriate antibiotics instead of the master plate. Selected clones were purified using the Zyppy Plasmid Miniprep kit (Zymo Research) and sequenced. Assembly of the sequences was performed using CodonCode Aligner software (CodonCode Corporation) with manual corrections.

## Additional Information

**Accession codes:** GenBank accession number KX279876. GenBank accession number KX359334.

**How to cite this article**: De Bustos, A. *et al*. Sequencing of long stretches of repetitive DNA. *Sci. Rep.* **6**, 36665; doi: 10.1038/srep36665 (2016).

**Publisher’s note:** Springer Nature remains neutral with regard to jurisdictional claims in published maps and institutional affiliations.
